# L-Arginine Mitigates Neutrophil-Induced Endothelial Injury by Preserving Nitric Oxide Signaling and Redox Homeostasis

**DOI:** 10.3390/ijms27188347

**Published:** 2026-09-19

**Authors:** Aurora Espejel-Nuñez, Arturo Flores-Pliego, Salvador Espino y Sosa, Johnatan Torres-Torres, Raigam Jafet Martinez-Portilla, Hector Borboa-Olivares, Araceli Montoya-Estrada, Aarón Galindo-Vega, Sandra Parra-Hernandez, Ismael Mancilla-Herrera, Juan Carlos Echeverria-Arjonilla, Ramon Gonzalez-Camarena, Guadalupe Estrada-Gutierrez

**Affiliations:** 1 Immunobiochemistry Department, Instituto Nacional de Perinatologia, Mexico City 11000, Mexico; aurora.espejel@inper.gob.mx (A.E.-N.); arturo.flores@inper.gob.mx (A.F.-P.); sandrabpahdz@gmail.com (S.P.-H.); 2 Postgraduate in Experimental Biology, Division of Biological and Health Sciences, Universidad Autónoma Metropolitana-Iztapalapa, Mexico City 09310, Mexico; 3 Department of Reproductive and Perinatal Health Research, Instituto Nacional de Perinatología, Mexico City 11000, Mexico; salvadorespino@gmail.com (S.E.y.S.); torresmmf@gmail.com (J.T.-T.); 4 Iberoamerican Research Network in Obstetrics, Gynecology and Translational Medicine, Mexico City, Mexico; raigam.martinez@gmail.com; 5 Clinical Research Branch, Instituto Nacional de Perinatologia, Mexico City 11000, Mexico; hector.borboa@inper.gob.mx; 6 Coordination of Gynecological and Perinatal Endocrinology, Instituto Nacional de Perinatologia, Mexico City 11000, Mexico; araceli.montoya@inper.gob.mx; 7 Functional Genomics of Cancer Laboratory, Instituto Nacional de Medicina Genomica, Mexico City 14610, Mexico; aarongave4648@gmail.com; 8 Biomedical Research Branch, Instituto Nacional de Perinatologia, Mexico City 11000, Mexico; mahi_25803@yahoo.com.mx; 9 Department of Electrical Engineering, Universidad Autónoma Metropolitana, Unidad Iztapalapa, Mexico City 09310, Mexico; jcea@xanum.uam.mx; 10 Department of Health Sciences, Universidad Autónoma Metropolitana, Unidad Iztapalapa, Mexico City 09310, Mexico; ramongoca@gmail.com

**Keywords:** endothelial dysfunction, oxidative stress, nitric oxide, L-arginine, neutrophils, redox signaling, antioxidant genes, endothelial repair, inflammation, vascular protection

## Abstract

Endothelial dysfunction is a hallmark of vascular injury and a central event in cardiovascular and inflammatory diseases. It is characterized by reduced nitric oxide (NO) bioavailability, increased oxidative stress, and disruption of endothelial barrier integrity. Activated neutrophils contribute to this process through the generation of reactive oxygen species and other oxidative mediators. Here, we investigated whether L-arginine, the principal substrate for endothelial NO synthase (eNOS), attenuates neutrophil-induced endothelial injury. Primary human umbilical vein endothelial cells (HUVECs) were exposed to sustained L-arginine supplementation initiated at passage 1 and subsequently challenged with activated neutrophils. Neutrophil exposure reduced stable NO metabolites (NOx), decreased eNOS phosphorylation at Ser1177, increased oxidant-sensitive DCF fluorescence, induced oxidative damage to lipids, proteins, and nucleic acids, disrupted PECAM-1/CD31-associated junctional organization, and increased endothelial permeability. Sustained L-arginine supplementation increased NOx accumulation in neutrophil-exposed cultures, partially preserved eNOS Ser1177 phosphorylation, attenuated oxidative damage, and improved endothelial barrier integrity. Catalase reduced oxidant-sensitive fluorescence and permeability, supporting a ROS-dependent component of the injury model. Targeted PCR array analysis identified neutrophil-associated changes in oxidative stress-related gene-expression patterns; however, direct comparison between L-arginine-supplemented and non-supplemented neutrophil-exposed cultures showed no statistically significant gene-expression changes after Benjamini–Hochberg FDR correction. These findings indicate that L-arginine attenuates neutrophil-induced endothelial injury primarily through biochemical, functional, and structural mechanisms, whereas transcriptional findings should be interpreted as exploratory and hypothesis-generating. Additional studies are required to determine the translational relevance of L-arginine supplementation as a potential approach to preserve endothelial function under oxidative and inflammatory stress.

## 1. Introduction

Endothelial dysfunction is an early hallmark of numerous cardiovascular, inflammatory, and metabolic diseases [1,2,3,4]. It is characterized by reduced nitric oxide (NO) bioavailability, increased oxidative stress, altered vascular tone, and enhanced leukocyte adhesion and infiltration [5,6]. Together, these alterations disrupt the tightly regulated mechanisms that maintain endothelial homeostasis, ultimately promoting vascular inflammation, thrombosis, and tissue hypoperfusion [7]. The capacity of endothelial cells to maintain or recover functional integrity following oxidative and inflammatory injury, known as endothelial resilience, has recently emerged as an important concept in vascular biology.

Neutrophils are critical mediators of endothelial oxidative and inflammatory injury [8,9,10]. Upon activation, these cells release large amounts of reactive oxygen species (ROS), proteases, myeloperoxidase, and neutrophil extracellular traps (NETs) that damage the endothelial barrier, induce cytotoxic injury, and promote redox imbalance [11,12,13,14,15]. ROS generated under these conditions rapidly react with NO, reducing its bioavailability and impairing endothelial nitric oxide synthase (eNOS) activity. Moreover, chronic oxidative stress may induce eNOS uncoupling, causing the enzyme to generate superoxide rather than NO, thereby amplifying oxidative stress and perpetuating endothelial dysfunction [16].

The semi-essential amino acid L-arginine is the major substrate for eNOS and a critical determinant of endothelial NO production [17]. Endothelium-derived NO is a potent vasodilator with anti-inflammatory, antithrombotic, and antioxidant properties, but its synthesis depends on adequate intracellular availability of L-arginine. Under oxidative stress, decreased L-arginine bioavailability and eNOS uncoupling further compromise NO production, aggravating endothelial dysfunction [18,19]. Reduced L-arginine availability has been linked to endothelial dysfunction in hypertension, metabolic disorders, chronic inflammation, and pregnancy-related complications [20,21,22]. In parallel, increased concentrations of asymmetric dimethylarginine (ADMA), a natural endogenous inhibitor of eNOS, compete with L-arginine and further suppress NO production [23]. Therefore, L-arginine supplementation has been proposed as a potential approach to improve NO-related homeostasis, preserve eNOS coupling, and attenuate oxidative endothelial injury. Importantly, emerging evidence indicates that the immunomodulatory effects of L-arginine are context-dependent. Previous studies have demonstrated gestational age-dependent effects on leukocyte recruitment and inflammatory responses, suggesting that L-arginine may also regulate leukocyte–endothelial interactions during inflammation [24].

Beyond serving as a substrate for NO synthesis, L-arginine may modulate redox-sensitive signaling pathways, including activation of the nuclear factor 2 (Nrf2)-antioxidant response element (ARE) pathway. This pathway regulates the transcription of antioxidant and cytoprotective enzymes, including superoxide dismutase (SOD), glutathione peroxidase (GPX), and heme oxygenase-1 (HMOX1) [25]. In addition to ROS detoxification, these antioxidant systems contribute to endothelial repair, cytoskeletal stabilization, and restoration of barrier integrity following inflammatory injury. Although increasing evidence supports a protective role for L-arginine in vascular homeostasis, the molecular mechanisms by which it counteracts neutrophil-induced endothelial dysfunction remain incompletely understood.

To address this knowledge gap, we established an in vitro coculture model with primary human umbilical vein endothelial cells (HUVECs) and activated neutrophils to reproduce acute inflammatory oxidative stress. We investigated whether L-arginine supplementation attenuates neutrophil-induced endothelial injury by influencing NO-related homeostasis, eNOS Ser1177 phosphorylation, redox balance, endothelial barrier integrity, and exploratory oxidative stress-related gene-expression patterns using complementary biochemical, structural, functional, and transcriptomic approaches. This study provides mechanistic insight into the vasoprotective actions of L-arginine during inflammatory stress and identifies exploratory oxidative-stress-related transcriptional patterns associated with endothelial responses. These findings provide mechanistic support for further investigation of L-arginine in more complex vascular models of inflammatory disease.

## 2. Results

### 2.1. Morphological and Phenotypic Characterization of HUVECs and Neutrophils

**Figure 1:**
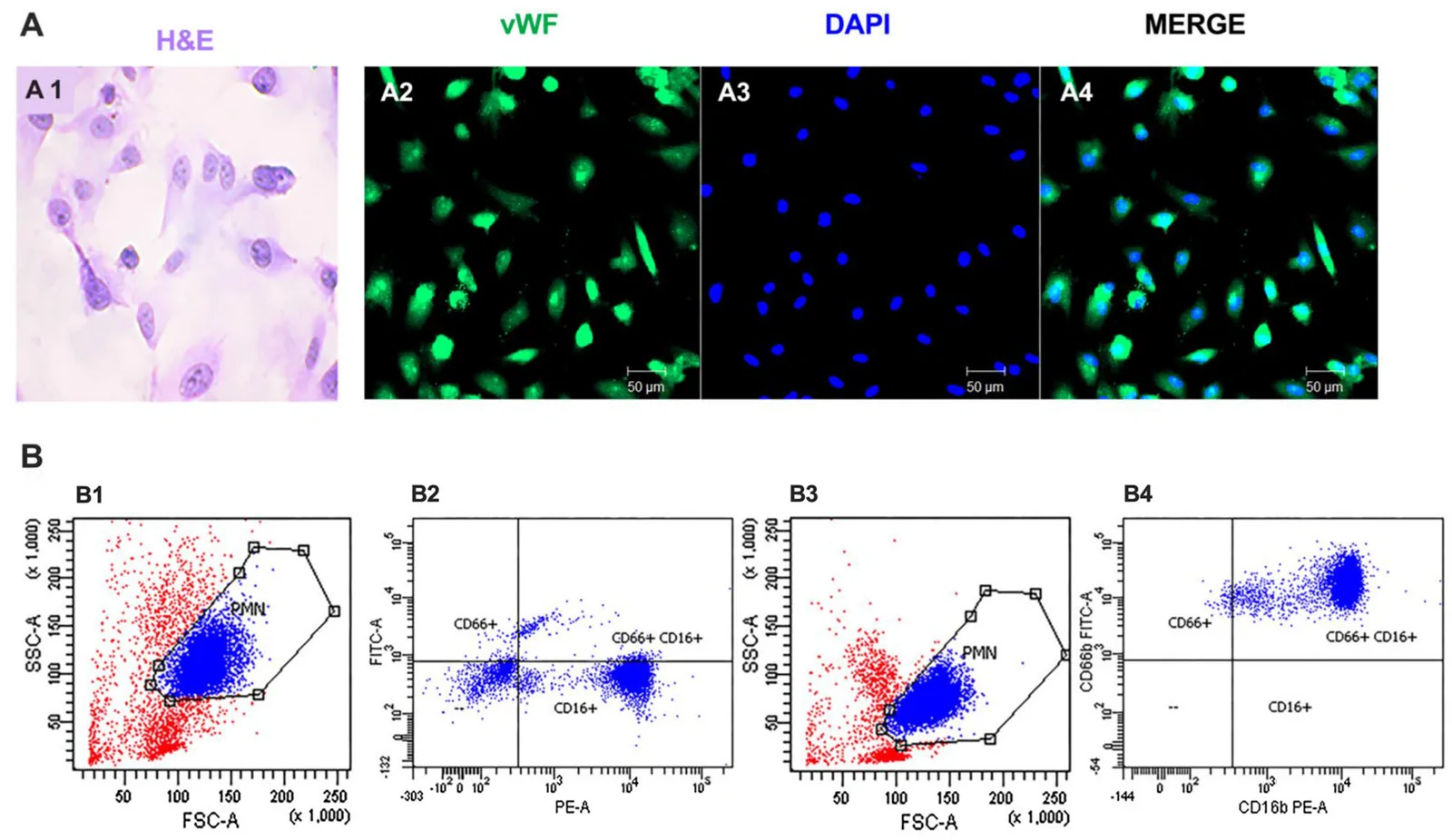
Characterization of isolated human umbilical vein endothelial cells (HUVECs) and neutrophils. (**A**) Representative images of primary HUVEC cultures: (**A1**) hematoxylin/eosin staining; (**A2**) Immunofluorescence staining for von Willebrand factor (vWF, green); (**A3**) DAPI nuclear counterstaining (blue); and (**A4**) merged image for vWF and DAPI signals. Scale bar 50 µm; 20× magnification. (**B**) Neutrophil phenotyping by flow cytometry: (**B1**,**B3**) Representative forward scatter (FSC) versus side scatter (SSC) density plots showing the gating strategy used to identify polymorphonuclear leukocytes (PMNs). (**B2**) Dot plot illustrating surface expression of CD16b+CD66b- in non-activated neutrophils. (**B4**) Activated neutrophils following arachidonic acid stimulation show marked upregulation of CD66b and reduced granularity, consistent with degranulation and a CD66b+CD16b+ phenotype. In FSC/SSC plots, blue events indicate the gated polymorphonuclear neutrophil population, whereas red events indicate events outside the selected PMN gate.

Primary HUVEC cultures were grown to confluence, forming a monolayer with the typical cobblestone morphology of endothelial cells. Endothelial identity was confirmed by positive immunofluorescence staining for von Willebrand factor (vWF), with homogeneous DAPI nuclear counterstaining (Figure 1(A2–A4)). These features were consistent with a well-differentiated endothelial phenotype and confirmed the suitability of the cultures for subsequent functional analyses.

Isolated neutrophils were phenotypically characterized by flow cytometry before coculture. Forward- and side-scatter analysis identified a predominant polymorphonuclear leukocyte population, with approximately 89% of events falling within the polymorphonuclear leukocyte gate (Figure 1(B1,B3)). Resting neutrophils displayed the expected CD16b-positive phenotype with low or absent CD66b signal (Figure 1(B2)), whereas arachidonic acid stimulation was associated with an apparent increase in CD66b expression and reduced side-scatter properties, consistent with activation-associated degranulation (Figure 1(B3,B4)). Together, these findings provide representative phenotypic quality-control evidence for the endothelial and neutrophil components used in the coculture model.

### 2.2. Activated Neutrophils Induce Oxidative Damage in Endothelial Cells

**Figure 2:**
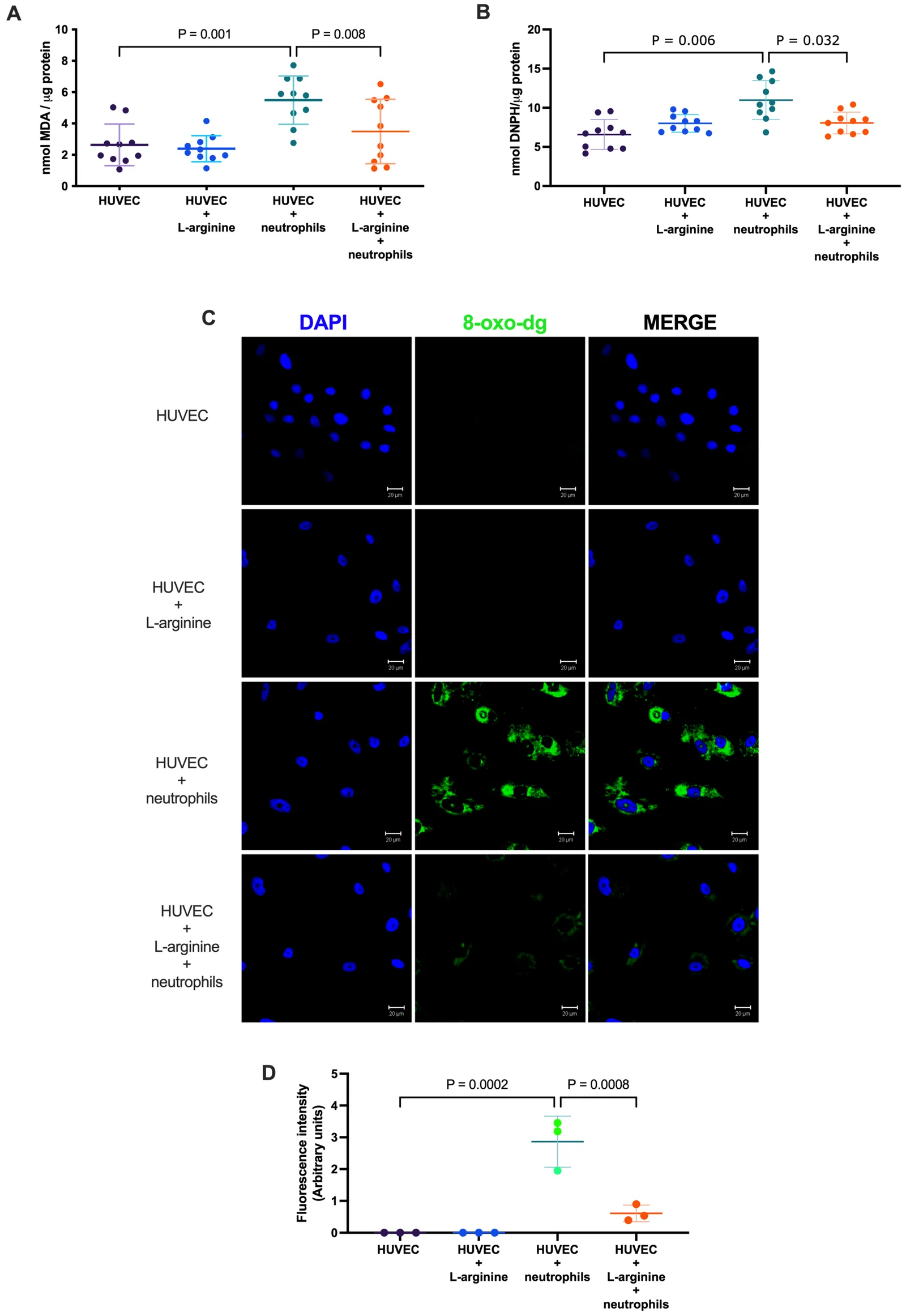
Activated neutrophils induce oxidative damage in endothelial cells. Quantification of oxidative stress-associated markers in HUVEC cultures under different experimental conditions: (**A**) Malondialdehyde (MDA) levels as an index of lipid peroxidation; (**B**) Protein carbonyl content determined by 2,4-dinitrophenylhydrazine (DNPH) derivatization. (**C**) Representative confocal immunofluorescence images of 8-hydroxy-2′-deoxyguanosine (8-oxo-dG; green), with DAPI nuclear counterstain (blue). 8-oxo-dG immunofluorescence was interpreted as evidence of oxidative nucleic acid damage. The predominantly cytoplasmic/perinuclear staining pattern does not allow assignment of the signal to a specific subcellular nucleic-acid compartment. (**D**) Densitometric analysis of 8-oxo-dG fluorescence intensity. Confocal images were acquired using a Zeiss Meta 510 laser-scanning confocal microscope (Carl Zeiss, Oberkochen, Germany) (40× magnification, scale bar: 20 µm). For panels A and B, data are presented as mean ± SD from ten independent HUVEC cultures and were analyzed using repeated-measures one-way ANOVA followed by Tukey’s multiple-comparison test. For panel (**D**), data are presented as mean ± SD from three independent HUVEC cultures; multiple microscopic fields were analyzed per condition but were not treated as independent biological replicates. Panel D was analyzed using ordinary one-way ANOVA followed by Tukey’s multiple-comparison test. Exact adjusted *p*-values for all pairwise comparisons are provided in Supplementary Table S3.

Exposure of HUVECs to activated neutrophils induced marked oxidative stress, as evidenced by increased oxidative damage to lipids, proteins, and nucleic acids (Figure 2). Lipid peroxidation, assessed by malondialdehyde (MDA), was significantly higher in neutrophil-exposed cells than in untreated controls (5.49 ± 1.54 vs. 2.63 ± 1.33 nmol MDA/µg total protein, *p* = 0.001) (Figure 2A). Protein oxidation was also significantly increased, as indicated by higher protein carbonyl levels in endothelial cells exposed to activated neutrophils compared with controls (11.00 ± 2.50 vs. 6.58 ± 1.91 nmol DNPH/µg total protein, *p* = 0.006) (Figure 2B).

Immunofluorescence analysis showed increased 8-oxo-2′-deoxyguanosine (8-oxo-dG) signal after neutrophil exposure (Figure 2C). Quantitative analysis confirmed a significant increase in 8-oxo-dG fluorescence intensity compared with untreated controls (*p* < 0.001) (Figure 2D). Although 8-oxo-dG is commonly used as a marker of oxidative nucleic acid damage, the staining pattern observed in our images was predominantly cytoplasmic/perinuclear rather than exclusively nuclear. This signal may reflect oxidized nucleic acid species in different cellular compartments, including a potential contribution from mitochondrial DNA or oxidized nucleotide pools. However, the present immunofluorescence approach does not allow determination of the precise subcellular source of the signal. Accordingly, these data are interpreted as evidence of oxidative nucleic-acid damage rather than as exclusive evidence of nuclear genomic oxidation.

L-arginine supplementation attenuated oxidative damage to lipids, proteins, and nucleic acids. Compared with cells exposed to activated neutrophils alone, L-arginine-supplemented cultures showed lower MDA levels (3.49 ± 2.06 vs. 5.49 ± 1.54 nmol MDA/µg protein, *p* = 0.008) and reduced 8-oxo-dG accumulation (*p* = 0.0008) (Figure 2A,C,D). Protein carbonyl levels were also significantly lower in cultures supplemented with L-arginine and exposed to neutrophils than in cultures exposed to neutrophils alone (*p* = 0.032; Figure 2B). Overall, these findings indicate that activated neutrophils induce substantial oxidative damage in endothelial cells and that L-arginine partially attenuates lipid, protein, and nucleic-acid oxidative damage.

### 2.3. Sustained L-Arginine Supplementation Increases NOx Accumulation and Partially Preserves eNOS Ser1177 Phosphorylation After Neutrophil Exposure

**Figure 3:**
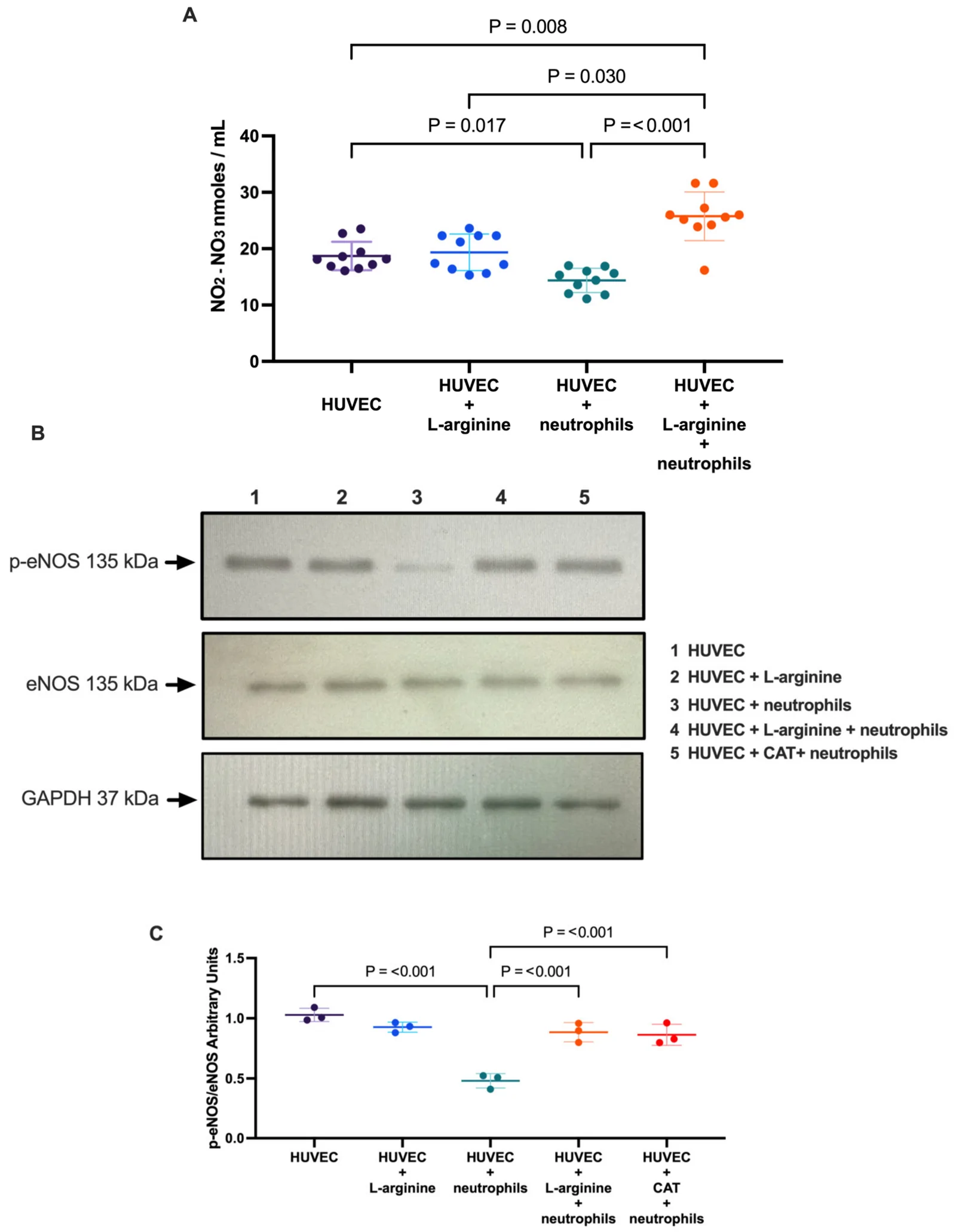
Sustained L-arginine supplementation increases NOx accumulation and partially preserves eNOS Ser1177 phosphorylation in neutrophil-exposed HUVECs. (**A**) Stable nitric oxide metabolites (nitrite and nitrate; NOx) in culture supernatants; (**B**) Representative immunoblots showing total endothelial nitric oxide synthase (eNOS) and phosphorylated eNOS at Ser1177 (p-eNOS). (**C**) Densitometric analysis of p-eNOS normalized to total eNOS. For panel A, data are presented as mean ± SD from ten independent HUVEC cultures and were analyzed using repeated-measures one-way ANOVA followed by Tukey’s multiple-comparison test. For immunoblot quantification in panels B and C, densitometric values were calculated from three independent HUVEC cultures and analyzed using ordinary one-way ANOVA followed by Tukey’s multiple-comparison test. Exact adjusted *p*-values for all pairwise comparisons are provided in Supplementary Table S3. For immunoblot quantification, total eNOS was normalized to GAPDH, whereas phosphorylated eNOS Ser1177 was normalized to total eNOS. Full uncropped immunoblot images, including molecular-weight markers and replicate blots, were uploaded as separate original/source image files through the journal submission platform. Densitometric values are provided in Supplementary Table S4.

Because NO is rapidly converted to nitrite and nitrate in aqueous biological systems, NO bioavailability was assessed indirectly by measuring stable NO metabolites, collectively referred to as NOx. Exposure to activated neutrophils significantly reduced NOx levels compared with untreated HUVECs (14.37 ± 2.16 vs. 18.72 ± 2.52 nmol NO_3_^−^/mL, *p* = 0.017), indicating impaired NO-related homeostasis under inflammatory oxidative stress. L-arginine supplementation increased NOx accumulation in neutrophil-exposed cultures compared with neutrophil exposure alone (25.75 ± 4.32 vs. 14.37 ± 2.15 nmol NO_3_^−^/mL, *p* < 0.001; Figure 3A). NOx levels in L-arginine-supplemented neutrophil-exposed cultures were also higher than those observed in untreated controls (*p* = 0.008), indicating that supplementation increased accumulated stable NO metabolites beyond baseline under neutrophil-exposure conditions. We also compared NOx levels between HUVECs supplemented with L-arginine alone and HUVECs supplemented with L-arginine and exposed to activated neutrophils; NOx levels were significantly different between these two conditions (*p* = 0.030), indicating that neutrophil exposure altered stable NO metabolite accumulation despite the presence of L-arginine. Because NOx represents cumulative nitrite/nitrate accumulation in the culture supernatant over the 24 h coculture period, rather than real-time NO production, this difference should not be interpreted solely as a direct reflection of endpoint p-eNOS Ser1177/eNOS status.

To determine whether the neutrophil-induced reduction in NOx was associated with changes in eNOS abundance and activation status in endothelial monolayer preparations after neutrophil exposure, total eNOS abundance and phosphorylation at the activating residue Ser1177 were assessed by immunoblotting. Total eNOS abundance did not differ markedly among experimental groups (Figure 3B,C), whereas neutrophil exposure reduced the p-eNOS Ser1177/eNOS ratio, consistent with decreased eNOS activation (Figure 3B,C).

L-arginine supplementation increased NOx accumulation and partially preserved the p-eNOS Ser1177/eNOS ratio in neutrophil-exposed cultures. These findings suggest that L-arginine was associated with maintenance of eNOS activation status and altered accumulation of stable NO metabolites under neutrophil-induced oxidative stress. However, this effect should not be interpreted as direct activation of eNOS phosphorylation by L-arginine.

### 2.4. L-Arginine and Catalase Reduce Oxidant-Sensitive DCF Fluorescence in Neutrophil-Exposed HUVECs

**Figure 4:**
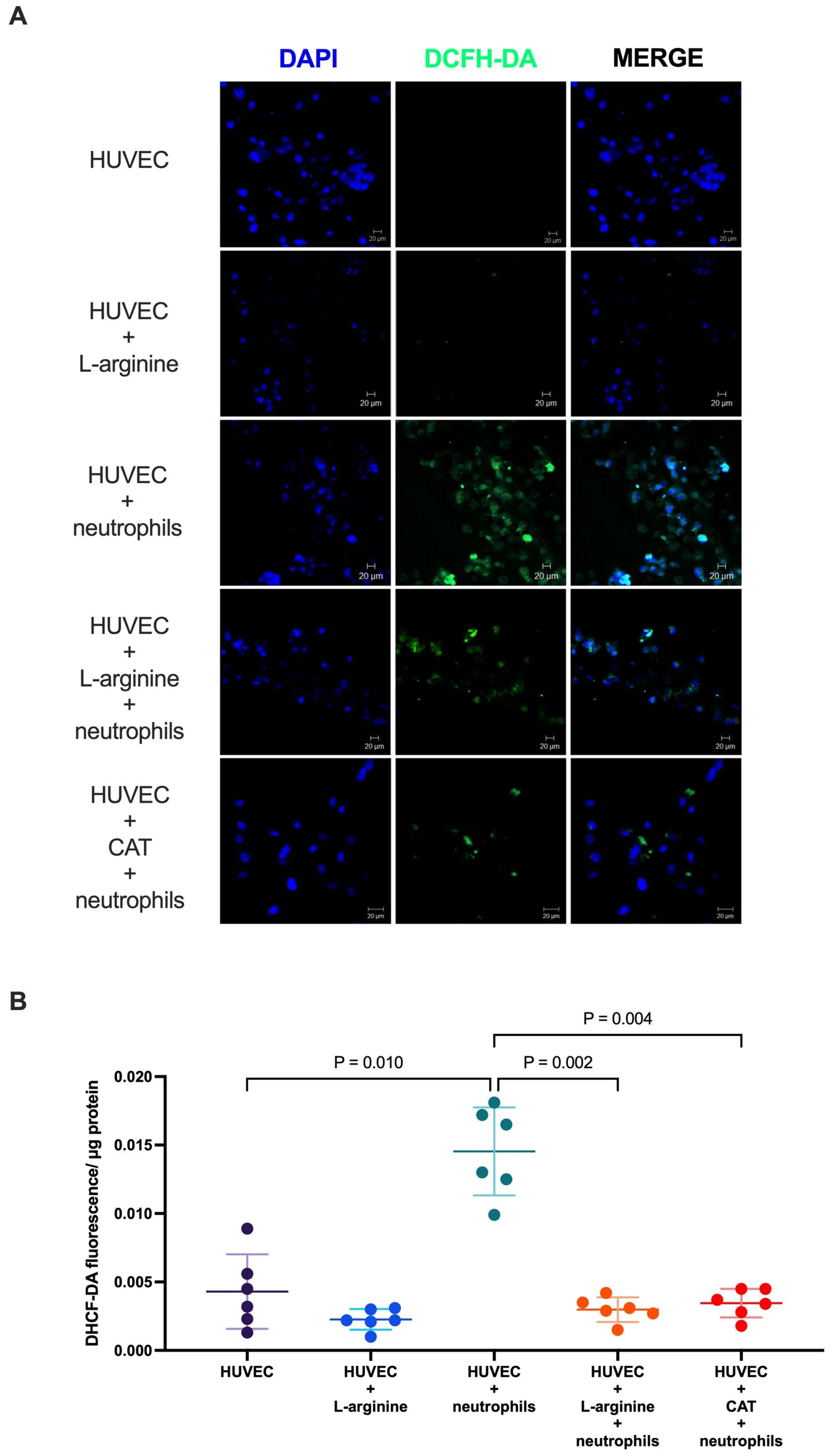
L-arginine and catalase reduce oxidant-sensitive DCF fluorescence in neutrophil-exposed HUVECs. (**A**) Representative confocal fluorescence microscopy images of DCFH-DA-derived fluorescence (green) in HUVECs with DAPI nuclear counterstaining (blue). (**B**) Quantification of intracellular oxidant-sensitive DCF fluorescence measured by DCFH-DA fluorescence in cell lysates and normalized to total protein content (µg). HUVECs were exposed to activated neutrophils in the presence or absence of L-arginine (200 µM) or catalase (500 U/mL). Catalase was added 30 min prior to neutrophil exposure and replenished after 12 h. Confocal images were acquired using a Zeiss Meta 510 laser-scanning microscope (20× magnification, scale bar 20 µm). Data are presented as mean ± SD from six independent HUVEC cultures. Technical replicates were averaged before statistical analysis. Data were analyzed using repeated-measures one-way ANOVA followed by Tukey’s multiple-comparison test. Exact adjusted *p*-values for all pairwise comparisons are provided in Supplementary Table S3.

Oxidant accumulation was assessed using the DCFH-DA assay. Because DCFH-DA does not identify a specific reactive oxygen species, results are reported as oxidant-sensitive DCF fluorescence rather than as direct quantification of a single ROS. Activated neutrophil exposure markedly increased oxidant-sensitive DCF fluorescence in HUVECs (0.0145 ± 0.0032 vs. 0.00430 ± 0.0027 IF/µg protein, *p* = 0.010) (Figure 4A,B), whereas L-arginine supplementation reduced this signal (*p* = 0.002). Catalase treatment also attenuated DCF fluorescence (*p* = 0.004), supporting the involvement of hydrogen peroxide-sensitive oxidative pathways in this model (Figure 4A,B).

### 2.5. Neutrophil-Induced Oxidative Stress Disrupts Endothelial Junctional Integrity and Increases Endothelial Permeability

**Figure 5:**
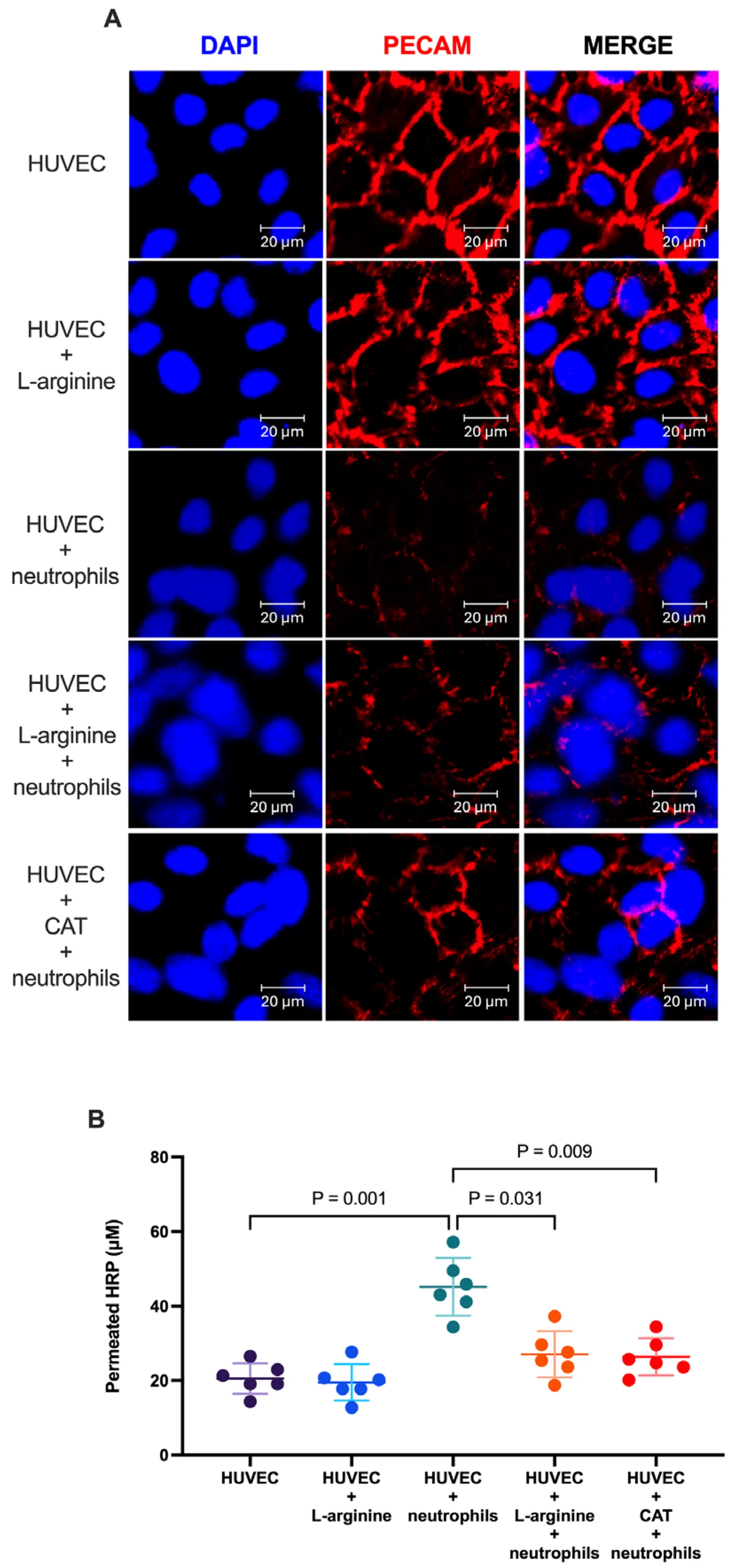
Neutrophil-induced endothelial dysfunction is associated with disruption of PECAM junctional integrity. (**A**) Representative confocal immunofluorescence images showing PECAM-1/CD31 localization (red) and DAPI nuclear counterstaining (blue) in HUVEC monolayers under the indicated experimental conditions. (**B**) Endothelial permeability assessed by the horseradish peroxidase (HRP) permeability assay. HUVECs were exposed to activated neutrophils in the presence or absence of L-arginine (200 µM) or catalase (500 U/mL). Catalase was added 30 min before neutrophil exposure and replenished after 12 h. Confocal images were acquired using a Zeiss Meta 510 laser-scanning microscope (20× magnification, scale bar 20 µm). PECAM-1/CD31 immunofluorescence images are representative of three independent HUVEC cultures and were used as qualitative structural support for endothelial junctional organization. Microscopic fields were not treated as independent biological replicates. HRP permeability data are presented as mean ± SD from six independent HUVEC cultures. HRP permeability data were analyzed using repeated-measures one-way ANOVA followed by Tukey’s multiple-comparison test. Exact adjusted *p*-values for all pairwise comparisons are provided in Supplementary Table S3.

To determine whether functional changes in endothelial permeability were accompanied by structural alterations in intercellular junctions, PECAM localization was evaluated by immunofluorescence. Under control conditions, PECAM exhibited a continuous and well-defined junctional staining pattern, consistent with intact endothelial monolayers (Figure 5A).

In contrast, exposure to activated neutrophils disrupted this junctional organization, resulting in discontinuous membrane staining, loss of cell–cell contacts, and the appearance of intercellular gaps (Figure 5A).

Representative images suggested partial preservation of junction-associated PECAM-1 staining in cultures supplemented with L-arginine or exposed to catalase.

Importantly, these structural changes were accompanied by a significant increase in endothelial permeability (45.18 ± 7.76 vs. 20.56 ± 4.10 µM permeated HRP, *p* = 0.001) as determined by the HRP permeability assay (Figure 5B), supporting the concept that neutrophil-induced barrier dysfunction involves both junctional remodeling and functional impairment. The protective effects of L-arginine and catalase further suggest that oxidative stress contributes to endothelial junctional instability and barrier dysfunction, as both interventions significantly reduced HRP permeability compared with neutrophil exposure alone (*p* = 0.031 and *p* = 0.009, respectively).

### 2.6. Neutrophil Exposure Alters Oxidative Stress-Related Gene Expression, with Exploratory Changes After L-Arginine Supplementation

To explore the transcriptional changes associated with neutrophil-induced oxidative stress and sustained L-arginine supplementation, we analyzed oxidative stress-related gene expression in endothelial monolayer preparations after coculture using an RT^2^ Profiler PCR Array Human Oxidative Stress Pathway (QIAGEN, Germantown, MD, USA). Because residual adherent neutrophils cannot be completely excluded after washing, these data were interpreted as exploratory gene-expression profiles of coculture-exposed endothelial monolayers rather than as purified endothelial transcriptomic responses.

**Figure 6:**
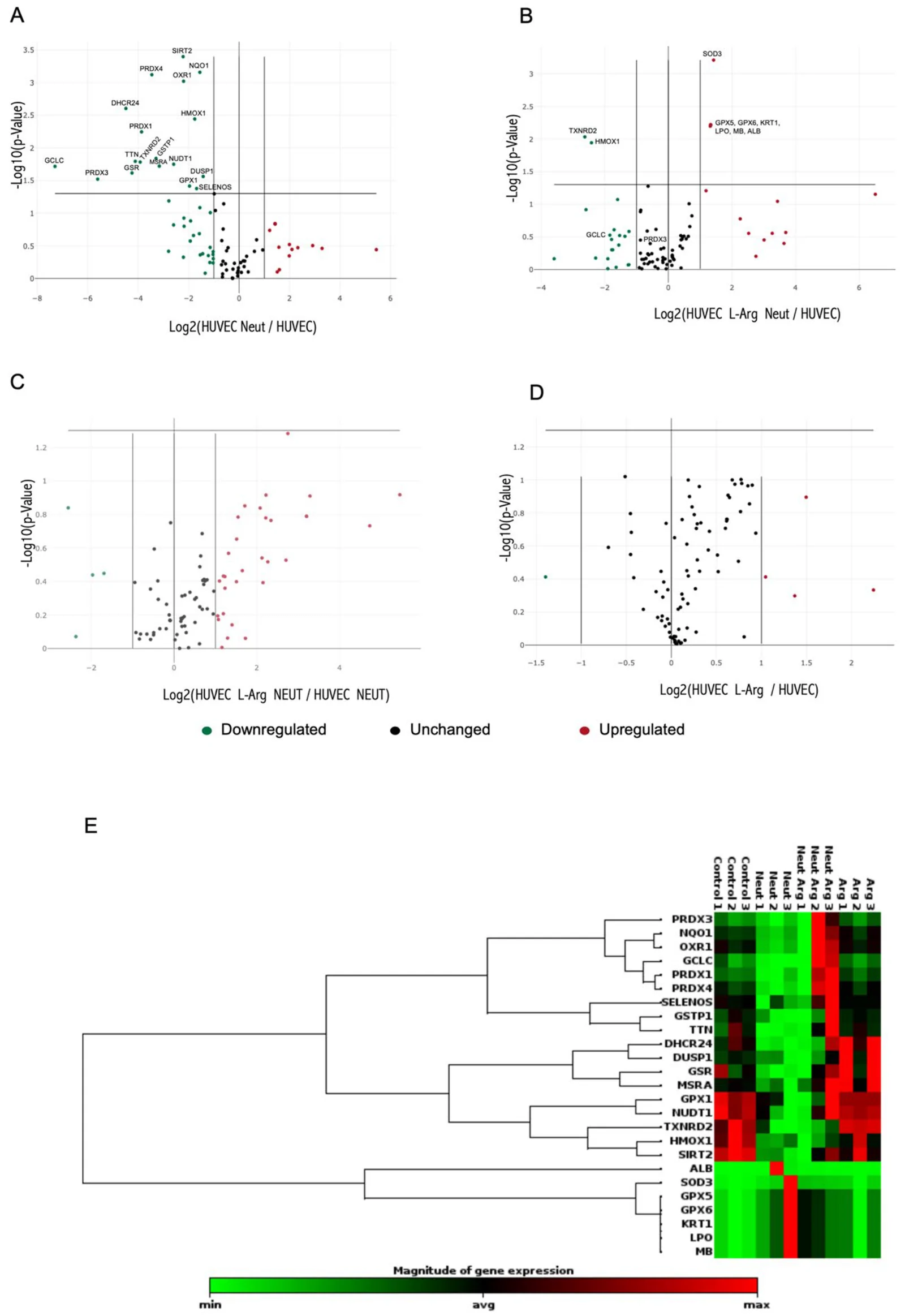
Transcriptional effects of L-arginine supplementation on oxidative stress–related genes in the endothelial dysfunction model. Volcano plots show differential expression of oxidative stress-related genes, with log_2_ fold change on the x-axis and -log_10_ raw *p*-value on the y-axis. The horizontal dashed line denotes the raw *p* < 0.05 threshold. Gene expression comparisons are shown for: (**A**) HUVECs exposed to neutrophils vs. control HUVECs; (**B**) HUVECs supplemented with L-arginine and exposed to neutrophils vs. control HUVECs; (**C**) HUVECs supplemented with L-arginine and exposed to neutrophils vs. neutrophil-exposed HUVECs; and (**D**) HUVECs supplemented with L-arginine alone vs. control HUVECs. (**E**) Exploratory clustergram based on genes selected using the nominal raw *p* < 0.05 threshold in at least one comparison before FDR correction. Because gene selection and clustering were performed using the same small PCR-array dataset, this visualization is descriptive and hypothesis-generating and should not be interpreted as independent evidence of group separation. Raw *p*-values were generated using the QIAGEN GeneGlobe two-tailed Student’s *t*-test and were additionally adjusted using the Benjamini–Hochberg false-discovery rate method. FDR-adjusted *p*-values are reported in Supplementary Table S2. These data should be interpreted as exploratory transcriptional profiling and not as definitive evidence of L-arginine-responsive antioxidant gene regulation. *n* = 3 independent HUVEC cultures per condition.

Using raw *p*-values, exposure of HUVECs to activated neutrophils was associated with changes in 18 antioxidant and redox-regulating genes (Figure 6A, Table 1). These included genes involved in glutathione biosynthesis, peroxide detoxification, thiol redox cycling, and mitochondrial redox regulation. After Benjamini–Hochberg FDR correction, only DHCR24, NQO1, OXR1, PRDX4, and SIRT2 remained statistically significant in the comparison between neutrophil-exposed HUVECs and untreated controls (Table 1).

L-arginine-supplemented cultures exposed to neutrophils showed increased expression of selected antioxidant-related genes compared with untreated control HUVECs, including SOD3, GPX5, GPX6, MB, ALB, LPO, and KRT1 (Figure 6B). However, these genes did not remain significant after FDR correction and were not significantly increased in the direct comparison between L-arginine-supplemented and non-supplemented neutrophil-exposed cultures (Figure 6C). Several genes downregulated after neutrophil exposure, including GCLC, PRDX3, GSR, PRDX1, PRDX4, and MSRA, showed positive fold-regulation trends after L-arginine supplementation, although none reached statistical significance after FDR correction. Therefore, PCR array results should be interpreted as exploratory modulation of oxidative stress-related gene-expression patterns rather than definitive evidence of L-arginine-responsive antioxidant gene regulation.

Importantly, L-arginine supplementation alone did not significantly alter gene expression in unstimulated HUVECs. Together with the absence of FDR-significant differences in the direct comparison between L-arginine-supplemented and non-supplemented neutrophil-exposed cultures, these findings support a cautious, exploratory interpretation of the transcriptional data (Figure 6D). An exploratory clustergram based on genes meeting the nominal raw *p* < 0.05 threshold in at least one comparison visually suggested group-associated expression patterns; however, because gene selection and clustering were performed using the same small PCR-array dataset, this visualization was considered descriptive and hypothesis-generating rather than independent statistical evidence of group separation (Figure 6E). Complete PCR array fold regulation values, raw *p*-values, and Benjamini–Hochberg FDR-adjusted *p*-values for all comparisons are provided in Supplementary Table S2.

## 3. Discussion

Endothelial dysfunction is a hallmark of inflammatory and vascular disorders and is characterized by reduced nitric oxide (NO) bioavailability, increased oxidative stress, and loss of endothelial barrier integrity [26,27]. In this study, we established an in vitro model of neutrophil-induced endothelial injury to investigate the protective effects of sustained L-arginine supplementation under conditions of acute oxidative and inflammatory stress. Our findings demonstrate that sustained L-arginine supplementation attenuates oxidant accumulation, limits oxidative damage to lipids, proteins, and nucleic acids, increases NOx accumulation, partially preserves eNOS Ser1177 phosphorylation, and improves endothelial barrier integrity. In contrast, PCR array findings support exploratory modulation of oxidative stress-related gene-expression patterns rather than definitive evidence of L-arginine-responsive antioxidant gene regulation.

Activated neutrophils are well-recognized mediators of vascular injury because they generate large amounts of reactive oxygen species (ROS), proteolytic enzymes, and pro-inflammatory mediators [9,12,28]. Consistent with previous reports, neutrophil exposure markedly increased intracellular oxidant-sensitive DCF fluorescence and oxidative damage while reducing NO metabolite levels, confirming the establishment of a profound redox imbalance [8,9]. The increase in 8-oxo-dG immunofluorescence further supports neutrophil-induced oxidative injury; however, the predominantly cytoplasmic/perinuclear staining pattern indicates that this signal should not be interpreted exclusively as nuclear genomic DNA damage. 8-oxo-dG immunoreactivity may include contributions from mitochondrial nucleic acids or oxidized nucleotide pools [29]; however, the present approach does not allow determination of the precise subcellular origin of the signal. We therefore interpret this endpoint more broadly as oxidative nucleic-acid damage. Although total eNOS protein abundance remained unchanged, phosphorylation at Ser1177 was significantly reduced, suggesting that the neutrophil-associated reduction in NOx levels was linked to impaired eNOS activation status rather than reduced protein expression. L-arginine increased NOx accumulation and partially preserved eNOS Ser1177 phosphorylation, supporting the concept that substrate availability contributes to maintenance of NO-related homeostasis and eNOS activation during inflammatory stress. It is important to note, however, that NOx and p-eNOS Ser1177/eNOS provide related but non-equivalent information. NOx reflects cumulative nitrite/nitrate accumulation over the 24 h coculture period, whereas p-eNOS Ser1177/eNOS represents the eNOS activation status at the time of protein extraction. Therefore, differences in NOx may also reflect NO consumption and oxidative conversion within the neutrophil-induced inflammatory redox environment and should not be interpreted as a direct one-to-one reflection of endpoint p-eNOS Ser1177/eNOS. L-arginine should not be interpreted as a direct activator of eNOS kinase activity. Rather, the partial preservation of eNOS phosphorylation at Ser1177 observed in neutrophil-exposed cultures likely reflects an indirect effect associated with attenuation of oxidative stress and preservation of redox-sensitive mechanisms regulating eNOS activation. Under neutrophil-induced oxidative stress, reduced NO bioavailability, increased oxidant burden, and disruption of endothelial homeostasis may impair eNOS activation. In this context, sustained L-arginine supplementation may help maintain NO-related homeostasis and partially preserve eNOS Ser1177 phosphorylation by supporting substrate availability and limiting oxidative injury. The absence of a marked increase in p-eNOS Ser1177 in unstimulated HUVECs suggests that L-arginine becomes functionally relevant primarily under inflammatory oxidative stress, when NO-related homeostasis and eNOS activation are compromised, rather than acting as a general stimulator of eNOS phosphorylation under basal conditions [30,31]. These observations are consistent with experimental and clinical evidence indicating that inflammatory conditions may render L-arginine availability rate-limiting for NO synthesis, particularly in the presence of elevated levels of asymmetric dimethylarginine (ADMA), an endogenous inhibitor of NOS [17,18,19,31]. Although ADMA was not measured in the present study, this mechanism provides a biologically plausible explanation for the observed maintenance of NO-related homeostasis and partial preservation of eNOS Ser1177 phosphorylation.

The protective effects of L-arginine extended beyond NO metabolism. Supplementation markedly reduced intracellular oxidant-sensitive DCF fluorescence accumulation together with lipid peroxidation, protein carbonylation, and oxidative nucleic-acid damage, indicating broad attenuation of oxidative injury. The inclusion of catalase as a functional ROS-scavenging control further supports the contribution of hydrogen peroxide-sensitive oxidative pathways to neutrophil-induced endothelial dysfunction. Catalase reduced intracellular oxidant-sensitive DCF fluorescence accumulation, improved endothelial barrier function, and partially preserved PECAM-1 junctional organization, consistent with a contribution of hydrogen peroxide and related ROS to endothelial barrier disruption. Likewise, L-arginine reduced endothelial permeability while preserving junctional integrity, linking the biochemical reduction in oxidative stress to the functional and structural preservation of endothelial integrity. Together, these findings support an important contributory role of oxidative stress in junctional remodeling and barrier dysfunction in this model.

These findings are also consistent with previous experimental studies demonstrating that L-arginine attenuates and regulates leukocyte–endothelial interactions by reducing leukocyte rolling, adhesion, and trans-endothelial migration under inflammatory conditions [24]. Although those studies focused primarily on leukocyte behavior rather than endothelial redox biology, together with our findings, they support a broader role for L-arginine in preserving vascular homeostasis by simultaneously limiting oxidative injury and modulating leukocyte–endothelial crosstalk during inflammation.

The PCR array findings should be interpreted with caution. Neutrophil exposure produced marked changes in oxidative stress-related gene-expression patterns, including genes involved in glutathione biosynthesis, peroxide detoxification, thiol redox homeostasis, and oxidative damage responses. In L-arginine-supplemented neutrophil-exposed cultures, selected antioxidant-associated genes showed raw *p*-value changes or positive fold-regulation trends; however, these changes did not remain significant after FDR correction in the direct comparison with untreated neutrophil-exposed cultures. Therefore, these data should be interpreted as exploratory modulation of redox-associated expression patterns rather than definitive evidence of L-arginine-responsive antioxidant gene regulation. Overall, the strongest evidence for L-arginine-mediated endothelial protection derives from biochemical, oxidative damage, NOx accumulation, eNOS Ser1177 phosphorylation, and barrier-function endpoints, whereas the PCR array provides exploratory transcriptional information requiring independent validation. Although activation of the Nrf2-antioxidant response element (ARE) pathway was not directly evaluated, the observed expression profile is consistent with Nrf2-mediated adaptive responses previously described in endothelial cells exposed to oxidative stress [32]. Beyond ROS detoxification, these antioxidant systems contribute to mitochondrial homeostasis, cytoskeletal organization, and maintenance of endothelial junctional integrity, processes that are fundamental for endothelial recovery following inflammatory injury [33].

The concept of endothelial resilience refers to the capacity of endothelial cells to restore functional homeostasis after acute injury [34]. Our findings suggest that L-arginine enhances this adaptive response through coordinated regulation of endothelial redox biology. Rather than acting solely as a substrate for eNOS, L-arginine appears to preserve endothelial function by simultaneously improving NO-related homeostasis, limiting oxidative molecular damage, and modulating exploratory oxidative stress-related gene-expression patterns. The convergence of these biochemical, structural, functional, and transcriptional effects supports a broader role for L-arginine in maintaining endothelial homeostasis during acute inflammatory stress.

**Figure 7:**
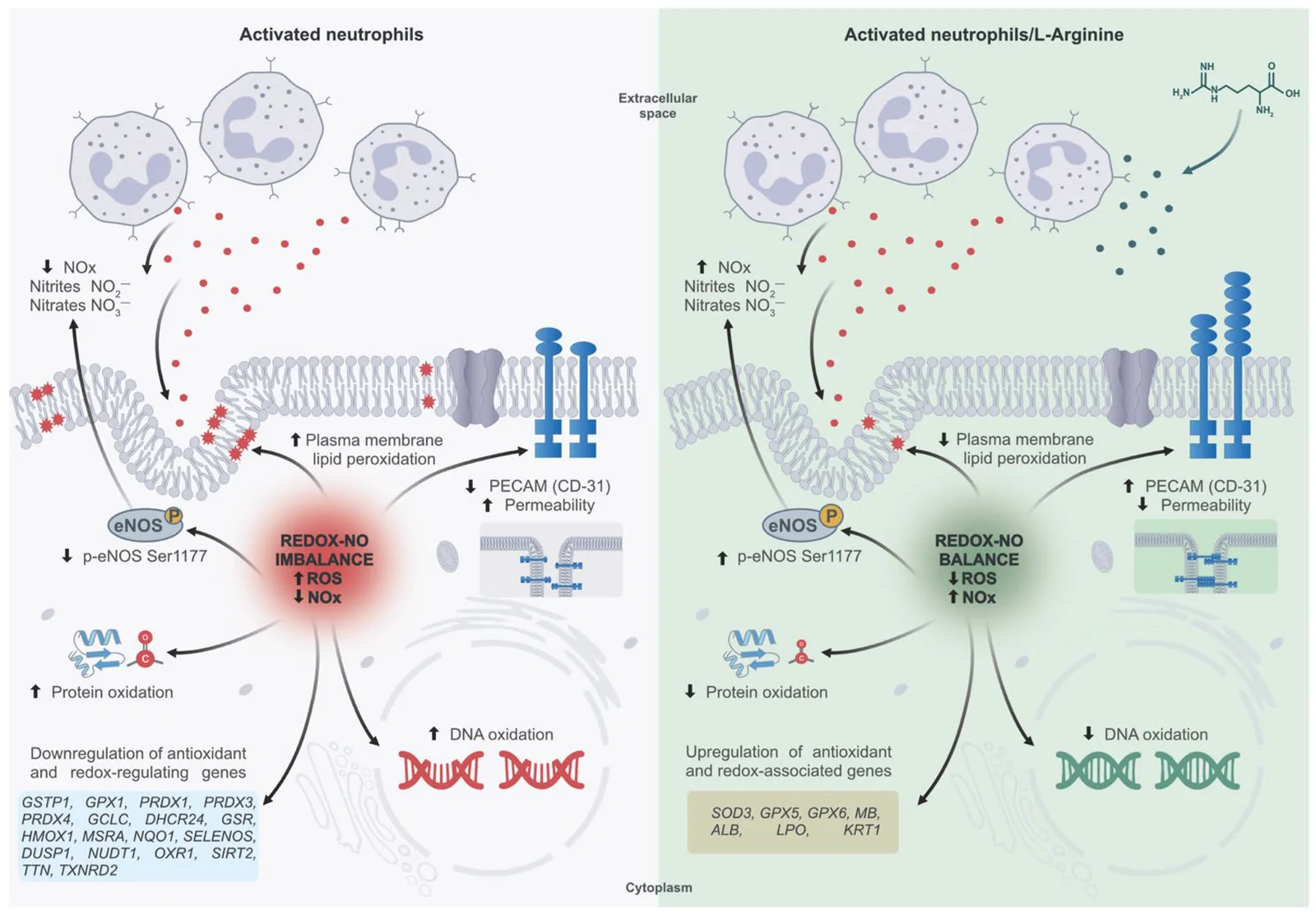
Proposed molecular model of L-arginine-mediated protection against neutrophil-induced endothelial dysfunction. Activated neutrophils promote endothelial injury through oxidant generation, leading to redox-NO imbalance, reduced NOx levels, impaired eNOS phosphorylation at Ser1177, lipid peroxidation, protein carbonylation, oxidative damage to nucleic acids, PECAM-1/CD31 junctional disorganization, and increased endothelial permeability. L-arginine attenuates this injury by increasing NOx accumulation, partially preserving eNOS Ser1177 phosphorylation, reducing oxidant-sensitive DCF fluorescence, limiting oxidative molecular damage, and improving endothelial barrier integrity. PCR array data suggest exploratory modulation of oxidative stress-related gene-expression patterns. The model integrates biochemical, structural, functional, and transcriptional findings and proposes that L-arginine enhances endothelial resilience against neutrophil-driven oxidative stress.

Based on these findings, we propose a mechanistic model in which activated neutrophils initiate endothelial dysfunction through ROS-mediated oxidative injury, resulting in reduced eNOS phosphorylation at Ser1177, reduced NOx accumulation and impaired NO-related homeostasis, disruption of PECAM-1 junctional organization, and increased endothelial permeability. L-arginine attenuates this injury pattern by increasing NOx accumulation, partially preserving eNOS Ser1177 phosphorylation, limiting oxidative damage to lipids, proteins, and nucleic acids, and modulating exploratory oxidative stress-related gene-expression patterns. These coordinated effects ultimately contribute to the preservation of endothelial barrier integrity. The reduction in ROS accumulation and endothelial permeability observed following catalase treatment further supports a ROS-dependent mechanism underlying neutrophil-induced endothelial dysfunction (Figure 7).

Our study has several strengths. It integrates complementary biochemical, functional, and transcriptomic approaches to characterize endothelial responses to neutrophil-induced injury. The use of primary HUVECs together with freshly isolated neutrophils provides a biologically relevant model of acute endothelial inflammation. Moreover, the simultaneous increase in NOx accumulation, partial preservation of eNOS Ser1177 phosphorylation, reduction in oxidative damage, improvement of endothelial barrier integrity, and exploratory modulation of oxidative stress-related gene expression patterns provide supportive, hypothesis-generating information.

Several limitations should also be acknowledged. First, HUVECs represent macrovascular endothelium, and endothelial responses may differ among vascular beds. Second, all experiments were performed under static culture conditions; therefore, the model does not account for shear stress, vascular flow, smooth muscle cell interactions, or multicellular vascular microenvironmental cues that may influence endothelial redox and barrier responses in vivo. Third, neutrophils were obtained from a single healthy donor and used as a standardized inflammatory stimulus. Although this approach reduced variability in neutrophil activation profiles, it does not capture interindividual heterogeneity in neutrophil oxidative burst, adhesion, degranulation, or transcriptomic contributions. Thus, the findings should be interpreted as HUVEC responses to a controlled neutrophil stimulus rather than as responses generalizable across neutrophil donors. In addition, although endothelial monolayers were washed before RNA and protein extraction, residual adherent neutrophils cannot be completely excluded and may have contributed to molecular readouts obtained after coculture. Accordingly, the immunoblot findings are interpreted primarily in the context of endothelial monolayer responses but cannot be considered exclusively endothelial in origin. Moreover, although L-arginine supplementation was initiated during endothelial culture expansion at passage 1, it remained present throughout the 24 h coculture period; therefore, potential direct effects on neutrophil oxidative burst, adhesion, degranulation, or activation marker expression cannot be completely excluded. Fourth, NO bioavailability was inferred from stable NO metabolites rather than measured in real time, and eNOS enzymatic activity, eNOS coupling status, tetrahydrobiopterin availability, and arginase activity were not directly assessed. Although total eNOS abundance and Ser1177 phosphorylation were evaluated, these measurements do not fully define eNOS activity or NO-generating capacity. Fifth, oxidant-sensitive DCF fluorescence does not identify a specific reactive oxygen species, and catalase was used as a functional ROS-scavenging control rather than as a complete characterization of the oxidative pathways involved. NET formation, inflammatory cytokine release, and Nrf2 activation were also not evaluated. Finally, the PCR array was performed with three independent HUVEC cultures per condition and should be interpreted as an exploratory, pathway-focused screening. After FDR correction, the direct comparison between L-arginine-supplemented and non-supplemented neutrophil-exposed cultures did not identify statistically significant L-arginine-responsive antioxidant gene regulation. Therefore, candidate transcriptional changes require confirmation by independent RT-qPCR in larger donor sets. Future studies using flow-based endothelial systems, organ-on-chip platforms, disease-specific endothelial models, and more selective approaches to distinguish specific ROS sources, including mitochondrial ROS production, and comparative studies of L-citrulline or L-arginine/ADMA interactions, will be required to provide greater mechanistic resolution before broader translational conclusions can be established [23,35,36].

Despite these limitations, the molecular mechanisms identified in this study are relevant to diseases characterized by endothelial dysfunction, oxidative stress and inflammatory activation, including atherosclerosis, metabolic disorders, sepsis, and pregnancy-related vascular complications. In particular, preeclampsia is characterized by systemic endothelial dysfunction, reduced NO availability, oxidative stress, and neutrophil activation [37,38,39]. Although our experimental model does not reproduce the complexity of pregnancy-specific vascular physiology, the observed effects of L-arginine on NO-related homeostasis, oxidative stress, and endothelial barrier function support further investigation in disease-relevant models [40,41,42].

In summary, our findings identify sustained L-arginine supplementation as a modulator of endothelial resilience during acute neutrophil-induced inflammatory stress. L-arginine increased NOx accumulation, partially preserved eNOS Ser1177 phosphorylation, limited oxidative injury, improved endothelial barrier integrity, and was associated with exploratory changes in oxidative stress-related gene-expression patterns. These findings support further evaluation of L-arginine as a candidate endothelial-protective approach in more complex vascular models of inflammatory diseases.

## 4. Materials and Methods

### 4.1. Ethics Statement

This study was approved by the Institutional Research, Ethics, and Biosafety Committee of the Instituto Nacional de Perinatologia Isidro Espinosa de los Reyes, in Mexico City (approval number 2017-3-118). All procedures were conducted in accordance with the principles of the Declaration of Helsinki for Medical Research Involving Human Subjects. Written informed consent was obtained from all participants before sample collection.

### 4.2. Study Subjects

Umbilical cords for HUVEC isolation were obtained during elective cesarean deliveries from 10 healthy pregnant women at term (39 ± 2 weeks of gestation). Gestational age was confirmed by first-trimester fetal ultrasound. The inclusion criteria were singleton pregnancy, absence of perinatal infection or maternal comorbidities (preeclampsia, obesity, diabetes mellitus, autoimmune diseases, uncontrolled thyroid disease, or renal/hepatic disease), and no use of medications known to affect endocrine or metabolic pathways (e.g., insulin, metformin, and corticosteroids).

HUVECs were obtained from ten independent umbilical cords. Because primary-cell yield varied among donors and different assays required varying numbers of cells, not all endpoints were performed on all ten donors. For each assay, all experimental conditions were tested in parallel within the same HUVEC donor whenever possible. The number of independent HUVEC donors included in each assay is specified in the corresponding figure legend and in Supplementary Table S1.

Neutrophils were isolated from peripheral venous blood collected from a single healthy, non-pregnant female with a normal body mass index and no recent history of illness. The use of a single donor was to minimize variability and allow consistency in the profiles of neutrophil activation.

### 4.3. Primary HUVEC Culture and Characterization

HUVECs were isolated from umbilical cords by a modified collagenase-free dissociation protocol, as previously described [43]. Umbilical veins were washed with PBS to eliminate remaining blood, then incubated with 1× trypsin-EDTA at 37 °C for 30 min. Cells were resuspended in EndoGRO-LS medium (Merck Millipore, Darmstadt, Germany) supplemented with 10% fetal bovine serum and antibiotics. Cells were cultured in 75 cm^2^ flasks at 37 °C in a humidified 5% CO_2_ incubator with medium changes every 48 h. Experiments were performed using cells at ~80% confluence and at passage 3 or earlier.

#### 4.3.1. Hematoxylin and Eosin Staining

Cell morphology was evaluated by hematoxylin/eosin (H&E) staining. Cells cultured on glass coverslips were fixed with 4% paraformaldehyde, washed with distilled water, and then stained with Harris hematoxylin to stain nuclei. Samples were counterstained with eosin after differentiation and bluing to detect cytoplasmic structures. Cells were dehydrated through a graded ethanol series; coverslips were then cleared in xylene and mounted using a permanent mounting medium. Images were collected with a bright-field microscope.

#### 4.3.2. Immunofluorescent Identification of HUVECs

The cells were fixed with 3.7% paraformaldehyde in PBS and permeabilized with 0.3% Triton X-100. Non-specific binding was blocked by incubation with human IgG for 20 min at room temperature. Endothelial cells were then incubated overnight at 4 °C in a humidified chamber with an anti-von Willebrand factor antibody (factor VIII) (Abcam, Cambridge, UK), conjugated to Alexa-488 (FITC) [44]. Cell nuclei were visualized by DNA staining using VECTASHIELD^®^ Vibrance™ antifade mounting medium containing 4′,6-diamidino-2-phenylindole (DAPI) (Vector Laboratories, Newark, CA, USA). Confocal images were acquired using a Zeiss Meta 510 laser-scanning microscope (Carl Zeiss Carl Zeiss, Oberkochen, Germany) under standardized acquisition parameters (488 nm excitation; 500–550 nm emission; 20× objective; pinhole 1.0 AU).

### 4.4. Neutrophil Isolation, Activation, and Flow Cytometry Characterization

Peripheral blood was collected in EDTA-K2 tubes and processed within one hour of collection. Neutrophils were isolated using Polymorphprep (Axis-Shield Diagnostics Ltd., Dundee, Scotland, UK), according to the manufacturer’s instructions, followed by erythrocyte lysis using 1× red blood cell lysis buffer (ThermoFisher Scientific, Carlsbad, CA, USA). Isolated polymorphonuclear (PMN) cells were washed twice and resuspended in 1× PBS.

#### 4.4.1. Neutrophil Activation Protocol

Neutrophils (1 × 10^6^ cells/mL) were stimulated with 50 µM arachidonic acid (Sigma-Aldrich, St. Louis, MO, USA) for 30 min at room temperature [45,46]. After activation, cells were washed and centrifuged at 500× *g* for 10 min to remove excess stimulant.

#### 4.4.2. Flow Cytometry Analysis

Neutrophils were fixed, permeabilized, and stained with titrated monoclonal antibodies against CD66b (FITC; clone G10F5, BD Pharmingen, San Diego, CA, USA) and anti-CD16b (PE; clone CLB-gran11.5, BD Pharmingen, San Diego, CA, USA). Data were acquired with a FACSAria III flow cytometer (BD Biosciences. San Jose, CA, USA). Compensation was performed using AbC™ Total Antibody Compensation beads (Invitrogen, Thermo Fisher Scientific, Carlsbad, CA, USA).

The gating strategy included: (i) Doublet exclusion (FSC-A vs. FSC-H); (ii) identification of polymorphonuclear cells based on FSC/SSC characteristics; (iii) identification of non-activated neutrophils (CD16b^+^/CD66b^−^); and (iv) identification of activated neutrophils (CD66b^+^/CD16b^+^). Flow cytometry was used as a representative phenotypic quality-control step to confirm neutrophil identity and activation-associated changes before coculture; however, quantitative MFI analysis, viability assessment, and repeated activation profiling were not systematically performed for all experiments.

### 4.5. In Vitro Endothelial Dysfunction Model

HUVECs were seeded in T-25 flasks and cultured to confluence. Activated neutrophils were added at a 16:1 neutrophil-to-HUVEC ratio and cocultured for 24 h at 37 °C in an atmosphere of 5% CO_2_ to induce an acute inflammatory oxidative injury model.

#### 4.5.1. L-Arginine Supplementation

After primary HUVEC cultures reached confluence, cells were passaged and separated into two parallel culture conditions: standard endothelial growth medium or endothelial growth medium supplemented with L-arginine. L-arginine (Sigma-Aldrich, St. Louis, MO, USA) was added to the endothelial growth medium at an additional concentration of 200 µM and was maintained during subsequent endothelial culture expansion and throughout the 24 h coculture period.

The reported 200 µM concentration refers to the amount of L-arginine experimentally added to the culture medium and does not include the basal L-arginine content already present in the endothelial growth medium. This study did not independently quantify the basal L-arginine concentration of the EndoGRO-LS medium (Merck Millipore, Darmstadt, Germany; catalog number SCME001). Therefore, the results should be interpreted as reflecting the effect of additional L-arginine supplementation during endothelial culture and neutrophil-induced challenge, rather than the effect of a defined total extracellular L-arginine concentration.

Concentration–response experiments were not performed; therefore, this study evaluated a single L-arginine supplementation condition. The selected concentration was consistent with physiological plasma and intracellular ranges and with evidence of adequate substrate availability for eNOS under inflammatory conditions [17,47,48].

#### 4.5.2. Experimental Groups

Four main experimental conditions were used: (1) control HUVECs cultured under standard conditions; (2) HUVECs supplemented with L-arginine from passage 1; (3) HUVECs exposed to activated neutrophils; and (4) HUVECs supplemented with L-arginine from passage 1 and subsequently exposed to activated neutrophils. For experiments evaluating ROS-dependent effects, additional catalase-treated conditions were included as described below.

Following incubation, culture supernatants were collected and centrifuged at 2000× *g* for 10 min. For protein extraction, fluorescence imaging, and RNA isolation, non-adherent neutrophils were first removed by aspirating the culture medium, and endothelial monolayers were gently washed three times with PBS before downstream processing. However, because activated neutrophils may adhere to endothelial cells, this procedure cannot completely exclude residual adherent neutrophils. Therefore, RNA and protein data obtained after coculture were interpreted as measurements from endothelial monolayer preparations exposed to neutrophils, with possible contribution from residual adherent neutrophils.

For experiments involving HUVECs, the independent biological unit was defined as the HUVEC donor when cultures were derived from different umbilical cords. For coculture experiments, neutrophils were obtained from a single healthy donor and used as a standardized inflammatory stimulus. Therefore, biological variability was captured primarily at the level of endothelial donors, whereas interindividual variability in neutrophil activation was not assessed. Technical replicates were averaged before statistical analysis.

#### 4.5.3. Catalase Supplementation

Catalase (500 U/mL) (Sigma-Aldrich. St. Louis, MO, USA) was added directly to the culture medium 30 min before neutrophil exposure and maintained throughout the 24 h coculture period. To preserve H_2_O_2_-scavenging activity during prolonged incubation, catalase was replenished once after 12 h. Catalase-treated cultures were included as a functional ROS-scavenging control to assess the contribution of hydrogen peroxide and related ROS to endothelial dysfunction.

### 4.6. Assessment of Oxidative Damage

#### 4.6.1. Protein Extraction

Cell lysates were obtained using M-PER™ reagent supplemented with Halt™ EDTA-free protease inhibitor cocktail (Pierce, Thermo Fisher Scientific, Rockford, IL, USA), according to the manufacturer’s instructions. Protein concentrations were determined using the bicinchoninic acid (BCA) method in a microplate format (Pierce, Thermo Fisher Scientific, Rockford, IL, USA).

#### 4.6.2. Lipid Peroxidation (MDA Assay)

Malondialdehyde (MDA) levels were quantified using the TBARS (TCA Method) Assay Kit (Cayman Chemical, Ann Arbor, MI, USA) according to the manufacturer’s instructions. In brief, cell lysates were mixed with thiobarbituric acid (TBA) reagent containing trichloroacetic acid (TCA) and incubated at 95 °C to permit production of MDA–TBA adducts. Samples were then cooled and centrifuged to remove precipitates. The supernatants were transferred to a 96-well plate, and absorbance was measured at 530–540 nm using a microplate spectrophotometer; MDA concentrations were determined from a standard curve built with known MDA equivalents. Results were normalized to total protein content and expressed as nmol MDA/µg protein.

#### 4.6.3. Protein Carbonylation (DNPH Assay)

Protein oxidation was assessed by measuring the protein carbonyl content by the Protein Carbonyl Colorimetric Assay Kit (Cayman Chemical, Ann Arbor, MI, USA), according to the manufacturer’s instructions. Briefly, cell lysates were incubated with 2,4-dinitrophenylhydrazine (DNPH) to convert carbonyl groups into stable hydrazone adducts. Samples were subsequently precipitated with trichloroacetic acid, washed to remove excess DNPH, and resuspended in guanidine hydrochloride. Absorbance was measured at 360–385 nm using a microplate spectrophotometer and expressed as nmol of carbonyl per µg of protein.

#### 4.6.4. Oxidative Nucleic-Acid Damage (8-oxo-dG)

Oxidative nucleic-acid damage was assessed by detecting 8-hydroxy-2′-deoxyguanosine (8-oxo-dG) using a monoclonal anti-8-oxo-dG antibody (Trevigen Inc., Gaithersburg, MD, USA) and counterstaining with DAPI. Cells were grown on glass coverslips, fixed with paraformaldehyde, permeabilized with Triton X-100, and incubated with primary antibody at 4 °C overnight. After washing, samples were incubated with an appropriate fluorophore-conjugated secondary antibody. Confocal images (20× magnification, 20 µm scale bars) were processed for densitometry using ImageJ software version 1.54p (National Institutes of Health, Bethesda, MD, USA).

#### 4.6.5. Stable Nitric Oxide Metabolites (NOx)

NO bioavailability was indirectly assessed by measuring stable nitric oxide metabolites, nitrite (NO_2_^−^) and nitrate (NO_3_^−^), collectively referred to as NOx. NOx levels were quantified in culture supernatants using a colorimetric assay (Cayman Chemical, Ann Arbor, MI, USA) according to the manufacturer’s protocol. Culture supernatants from the experimental groups were incubated with nitrate reductase and its cofactor, after which the Griess reagent was added and incubated for 15 min. Absorbance was then recorded at 540 nm using a Synergy™ HT microplate reader (BioTek Instruments, Inc., Winooski, VT). Because this assay measures accumulated stable NO metabolites rather than real-time NO generation, the results are reported as NOx levels and interpreted as an indirect readout of NO-related homeostasis.

#### 4.6.6. Immunoblot Analysis of eNOS and Phosphorylated eNOS

Total protein extracts were obtained from HUVECs using M-PER™ reagent supplemented with Halt™ EDTA-free protease inhibitor cocktail. Equal amounts of protein were resolved by SDS-PAGE gels at 8% and transferred onto PVDF membranes. Membranes were blocked with 5% non-fat dry milk or bovine serum albumin and incubated overnight at 4 °C with primary antibodies against total endothelial nitric oxide synthase (eNOS) (Abcam, Cambridge, UK; Cat. No. ab199956; dilution 1:1000), and phosphorylated eNOS at Ser1177 (Cell Signaling Technology, Danvers, MA, USA; Cat. No. 9571; dilution 1:1000). Membranes were then incubated with secondary antibody, anti-rabbit–HRP, dilution 1:5000 (Abcam, Cambridge, UK; Cat. No. 6721). Membranes were developed using the SuperSignal™ West Femto Maximum Sensitivity Substrate (Thermo Scientific Pierce, Rockford, IL, USA; cat. 34096) and visualized on Amersham Hyperfilm™ ECL photographic films (GE Healthcare, UK Limited, Little Chalfont, Buckinghamshire, UK; cat. 28906835). Band intensities were quantified by densitometry using ImageJ. Total eNOS was normalized to anti-GAPDH antibody (Abcam, Cambridge, UK; Cat. No. 8245; dilution 1:5000), whereas p-eNOS Ser1177 was normalized to total eNOS. Densitometric values were calculated from *n* = 3 independent HUVEC cultures and are presented relative to the untreated control group. Full uncropped immunoblot images, including molecular-weight markers and replicate blots, were uploaded as separate original/source image files through the journal submission platform. The corresponding densitometric values are provided in Supplementary Table S4.

### 4.7. Quantification of Oxidant-Sensitive DCF Fluorescence Using DCFH-DA

Oxidant-sensitive fluorescence was assessed using 2′,7′-dichlorofluorescin diacetate (DCFH-DA) (Sigma-Aldrich, St. Louis, MO, USA). As previously described [49], HUVECs were treated with DCFH-DA (10 µM) and incubated for 30 min at 37 °C. Following incubation, cells were washed thoroughly to remove excess probe, and fluorescence was quantified in protein-normalized lysates using a microplate reader (excitation 485 nm; emission 530 nm). Concomitantly, treated cells seeded on coverslips were fixed and processed for confocal microscopy to assess oxidant-sensitive DCF fluorescence, with similar acquisition settings across experimental conditions. Fluorescence was measured by ImageJ software.

### 4.8. Endothelial Barrier Function (HRP Permeability Assay)

HUVECs were cultured on polycarbonate membrane inserts (24-well format). Horseradish peroxidase (0.5 mM) (Sigma-Aldrich, St. Louis, MO, USA) was added to the upper chamber, and after 1 h, HRP activity in the lower chamber was quantified at 470 nm using a spectrophotometer as previously described [50]. To complement the functional assessment of endothelial permeability, PECAM-1/CD31 immunofluorescence was performed to evaluate junctional organization and structural integrity of endothelial cell–cell contacts.

### 4.9. PECAM-1 Immunofluorescence Analysis

Endothelial junctional organization was evaluated by immunofluorescent detection of platelet endothelial cell adhesion molecule-1 (PECAM-1/CD31) using an Alexa Fluor 647-conjugated antibody (Abcam, Waltham, MA, USA; Cat. No. Ab218582). HUVECs grown on glass coverslips were subjected to the indicated experimental conditions, washed with PBS, and fixed with 4% paraformaldehyde for 15 min at room temperature. The cells were permeabilized in PBS with Triton X-100 and blocked with bovine serum albumin to prevent non-specific binding. Samples were incubated overnight with a primary antibody against PECAM-1/CD31 (1:500 dilution) at 4 °C. Coverslips were mounted using antifade mounting medium, and nuclei were counterstained with DAPI. Images were acquired using a laser-scanning confocal microscope under identical acquisition settings for all experimental groups. PECAM-1 junctional organization was qualitatively assessed based on the continuity of membrane-associated staining and the presence of intercellular gaps.

### 4.10. Oxidative Stress-Focused Gene Expression Profiling

Total RNA was extracted from HUVECs under the different experimental conditions using the RNAqueous™ Total RNA Isolation Kit (Ambion/Invitrogen, Thermo Fisher Scientific, Carlsbad, CA, USA). RNA concentration was determined using a NanoDrop 2000 (Thermo Fisher Scientific, Wilmington, DE, USA), and RNA integrity was assessed using an Agilent 2100 Bioanalyzer (Agilent Technologies, Santa Clara, CA, USA). cDNA was synthesized using the RT^2^ First Strand kit (QIAGEN, Germantown, MD, USA). The gene expression profiles were determined by the RT^2^ Profiler™ PCR Array Human Oxidative Stress Pathway (QIAGEN Germantown, MD, USA), which contains 84 genes involved in antioxidant defenses, ROS metabolism, and oxidative regulation.

The reactions were run in SYBR Green Master Mix (QIAGEN, Germantown, MD, USA) on an Applied Biosystems™ StepOnePlus™ Real-Time PCR System (Applied Biosystems, Foster City, CA, USA). Data were analyzed using the 2^−ΔΔCt^ method and normalized to five housekeeping genes (ACTB, B2M, GAPDH, HPRT1, and RPLP). Expression analyses were carried out in biological triplicate.

### 4.11. Statistical Analyses

No formal a priori sample-size calculation was performed because this study was designed as a mechanistic in vitro study using primary endothelial cultures rather than as a clinical or epidemiological study powered for a predefined clinical endpoint. The number of independent experiments was determined by the availability and yield of primary HUVEC cultures, the requirement to test all experimental conditions in parallel, whenever possible, and the feasibility of performing multiple biochemical, functional, structural, and molecular endpoints from donor-derived cells.

The experimental design prioritized assessing biologically consistent endothelial responses to a standardized neutrophil-induced inflammatory stimulus across independent HUVEC cultures. For the RT^2^ Profiler PCR Array, three independent cultures per condition were included as an exploratory pathway-focused analysis. This design allowed statistical comparison within the GeneGlobe analysis workflow; however, given the limited number of biological replicates and the number of genes evaluated, PCR array findings were interpreted as hypothesis-generating. Therefore, raw *p*-values were additionally evaluated using Benjamini–Hochberg false-discovery rate correction, and transcriptional results were not used as standalone evidence of definitive L-arginine-responsive gene regulation.

Data distribution was assessed using the Shapiro–Wilk test, and homogeneity of variance was evaluated using the Brown–Forsythe test. These assumption checks were interpreted cautiously, particularly for endpoints evaluated with only three independent HUVEC cultures, because of the limited reliability of normality and variance testing in very small datasets. Individual biological data points are shown in the corresponding figures whenever applicable. Because experimental conditions within most quantitative endpoints were generated in parallel from the same HUVEC donor IDs whenever possible, donor-matched analyses were applied when the final endpoint-level data structure supported this approach. MDA, protein carbonyl content, NOx, oxidant-sensitive DCF fluorescence, and HRP permeability were analyzed using repeated-measures one-way ANOVA followed by Tukey’s multiple-comparison test. For 8-oxo-dG immunofluorescence and immunoblot densitometry, which were evaluated in n = 3 independent HUVEC cultures and yielded endpoint-level summary values from image-field or densitometric analyses, ordinary one-way ANOVA followed by Tukey’s multiple-comparison test was used; these endpoints were interpreted cautiously. Technical replicates were averaged before statistical analysis and were not treated as independent biological replicates. Values are expressed as mean ± SD. A *p*-value < 0.05 was considered statistically significant. The statistical test used for each endpoint, biological replicate number, matching structure, and exact pairwise *p*-values are summarized in Supplementary Table S3.

PCR array data were initially analyzed using QIAGEN GeneGlobe Data Analysis Center (https://geneglobe.qiagen.com/us/analyze, accessed on 6 July 2023), according to the manufacturer’s workflow. Fold regulation values were calculated using the 2^−ΔΔCt^ method, and raw *p*-values were generated by two-tailed Student’s *t*-test applied to replicate 2^−ΔCt^ values for each gene. To account for multiple testing across the oxidative stress-focused gene panel, raw *p*-values were additionally adjusted using the Benjamini–Hochberg false-discovery rate method. FDR adjustment was applied separately to each experimental comparison across the 84 target genes included in the array, excluding housekeeping genes and technical controls. Both raw and FDR-adjusted *p*-values are provided in Supplementary Table S2. Genes with raw *p* < 0.05 but FDR-adjusted *p*-values greater than or equal to 0.05 were interpreted as exploratory transcriptional changes rather than definitive differentially expressed genes. According to the software’s specifications, *p*-values are generated only when each experimental group contains at least three biological replicates, and statistically significant differences (*p* < 0.05) are highlighted by the system.

## 5. Conclusions

The present study shows that sustained L-arginine supplementation attenuates neutrophil-induced endothelial injury under controlled in vitro conditions. L-arginine increased NOx accumulation in neutrophil-exposed cultures, partially preserved eNOS phosphorylation at Ser1177, reduced oxidant-sensitive DCF fluorescence, limited oxidative damage to lipids, proteins, and nucleic acids, and improved endothelial barrier integrity. The inclusion of catalase further supports a ROS-dependent component of neutrophil-induced endothelial dysfunction.

Targeted PCR array analysis identified oxidative stress-related gene-expression changes after neutrophil exposure. However, the direct comparison between L-arginine-supplemented and non-supplemented neutrophil-exposed cultures did not show statistically significant changes in gene expression after FDR correction. Therefore, transcriptional findings should be interpreted as exploratory and hypothesis-generating rather than as definitive evidence of L-arginine-responsive antioxidant gene regulation.

Although this model does not fully recapitulate the complexity of vascular physiology, including flow-dependent signaling, smooth muscle interactions, NET formation, or systemic inflammatory responses, it provides mechanistic insight into early endothelial responses to neutrophil-mediated oxidative injury. These findings support further evaluation of L-arginine in multicellular, flow-based, disease-specific, and in vivo models before therapeutic conclusions can be established.

## Figures and Tables

**Table 1:** Summary of FDR-significant oxidative stress-related PCR array findings.

Comparison	Raw *p* < 0.05	FDR < 0.05	FDR-Significant Genes	Interpretation
HUVEC + neutrophils vs. HUVEC	18	5	DHCR24, NQO1, OXR1, PRDX4, SIRT2	Neutrophil exposure induced significant redox-associated transcriptional changes after FDR correction.
HUVEC + L-arginine + neutrophils vs. HUVEC	9	0	None	Changes were not significant after FDR correction and should be interpreted as exploratory.
HUVEC + L-arginine + neutrophils vs. HUVEC + neutrophils	0	0	None	No statistically significant L-arginine-responsive antioxidant gene regulation was detected in the direct comparison after FDR correction.
HUVEC + L-arginine vs. HUVEC	0	0	None	L-arginine alone did not induce significant oxidative stress-related transcriptional changes.

After FDR correction, DHCR24, NQO1, OXR1, PRDX4, and SIRT2 remained significant in the comparison between neutrophil-exposed HUVECs and untreated HUVECs. In contrast, no gene reached statistical significance in the direct comparison between L-arginine-supplemented and non-supplemented neutrophil-exposed cultures.

## Data Availability

The data sets used and/or analyzed during the current study are available from the corresponding author upon reasonable request.

## Supplementary Materials

The following supporting information can be downloaded at: https://www.mdpi.com/article/10.3390/ijms27188347/s1.
