# Supplementary material for: L-Arginine Mitigates Neutrophil-Induced Endothelial Injury by Preserving Nitric Oxide Signaling and Redox Homeostasis
Source: Int J Mol Sci. 2026 Sep 19;27(18):8347. doi: 10.3390/ijms27188347 (PMC13607857; doi:10.3390/ijms27188347)
Supplement: Supplementary file 1 [file ijms-27-08347-s001.zip › Supplementary_Table_S1_HUVEC_Donor_Distribution.pdf]

Supplementary Table S1. HUVEC donor distribution across assays and biological replicate definition					
**Assay / endpoint**	**Figure**	**Number of independent HUVEC cultures / donors**	**HUVEC donor IDs included**	**Matching status**	**Technical replicate handling**
HUVEC morphology and vWF characterization	Figure 1A	n = 3	HUVEC_01-HUVEC_03	Not applicable / characterization assay	Representative images; technical fields were not treated as independent biological replicates.
Neutrophil phenotypic characterization by flow cytometry	Figure 1B	Neutrophils from one donor	Neutrophil preparation from a single donor	Not applicable; single neutrophil donor, resting vs activated neutrophils	Used as representative phenotypic quality-control data.
Lipid peroxidation / MDA	Figure 2A	n = 10	HUVEC_01-HUVEC_10	Fully matched	Technical replicates were averaged before analysis.
Protein carbonyl content	Figure 2B	n = 10	HUVEC_01-HUVEC_10	Fully matched	Technical replicates were averaged before analysis.
8-oxo-dG immunofluorescence	Figure 2C,D	n = 3	HUVEC_01, HUVEC_03, HUVEC_07	Fully matched	Microscopic fields were not treated as independent biological replicates.
Stable nitric oxide metabolites / NOx	Figure 3A	n = 10	HUVEC_01-HUVEC_10	Fully matched	Technical replicates were averaged before statistical analysis.
Total eNOS and p-eNOS Ser1177 immunoblotting	Figure 3B,C	n = 3	HUVEC_06, HUVEC_08, HUVEC_10	Fully matched	Densitometric values were calculated per independent culture; technical measurements were averaged.
Oxidant-sensitive DCF fluorescence	Figure 4	n = 6	HUVEC_03, HUVEC_05, HUVEC_07-HUVEC_10	Fully matched	Technical replicates were averaged before statistical analysis.
Catalase rescue assay / DCF fluorescence	Figure 4	n = 6	HUVEC_03, HUVEC_05, HUVEC_07-HUVEC_10	Fully matched	Technical replicates were averaged before statistical analysis.
PECAM-1/CD31 immunofluorescence	Figure 5A	n = 3	HUVEC_08-HUVEC_10	Qualitative endpoint; not included in statistical matching	Representative images; microscopic fields were not treated as independent biological replicates.
HRP permeability assay	Figure 5B	n = 6	HUVEC_03, HUVEC_05, HUVEC_07-HUVEC_10	Fully matched	Technical replicates were averaged before statistical analysis.
RT² Profiler PCR array	Figure 6	n = 3	HUVEC_01, HUVEC_03, HUVEC_07	Fully matched; exploratory screening	GeneGlobe analysis was performed using three independent HUVEC cultures per condition; transcriptional findings were interpreted as exploratory.

Note: HUVECs were obtained from ten independent umbilical cords. For each quantitative endpoint, the listed HUVEC donor IDs indicate the independent biological replicates included in the analysis. "Fully matched" indicates that all experimental conditions compared within that endpoint were tested in parallel using the same HUVEC donors. Technical replicates and microscopic fields were averaged or used for representative analysis before statistical comparison and were not considered independent biological replicates. For coculture experiments, neutrophils were obtained from a single healthy donor and used as a standardized inflammatory stimulus; therefore, biological variability was captured primarily at the endothelial donor level. PECAM-1/CD31 immunofluorescence was assessed qualitatively and was not included as a quantitative statistical endpoint.
