# Supplementary material for: L-Arginine Mitigates Neutrophil-Induced Endothelial Injury by Preserving Nitric Oxide Signaling and Redox Homeostasis
Source: Int J Mol Sci. 2026 Sep 19;27(18):8347. doi: 10.3390/ijms27188347 (PMC13607857; doi:10.3390/ijms27188347)
Supplement: Supplementary file 1 [file ijms-27-08347-s001.zip › Supplementary_Table_S3_Statistical_Tests_and_Pvalues.pdf]

Supplementary Table S3. Statistical tests, biological replicates, matching structure, and exact p-values for quantitative endpoints

Figure / panel	Endpoint	Biological n	Technical replicate handling	Assumption testing	Omnibus test	Post hoc / pairwise test	Exact p-values / pairwise comparisons
Figure 2A	Lipid peroxidation / MDA	n = 10 independent HUVEC cultures	Technical replicates averaged before statistical analysis	Shapiro–Wilk; Brown–Forsythe	Repeated-measures one-way ANOVA	Tukey's multiple-comparison test	Neutrophils vs control: p = 0.001; L-arginine + neutrophils vs control: p = 0.726; L-arginine vs control: p = 0.964; L-arginine + neutrophils vs neutrophils: p = 0.008; L-arginine vs neutrophils: p < 0.001; L-arginine + neutrophils vs L-arginine: p = 0.353
Figure 2B	Protein carbonyl content	n = 10 independent HUVEC cultures	Technical replicates averaged before statistical analysis	Shapiro–Wilk; Brown–Forsythe	Repeated-measures one-way ANOVA	Tukey's multiple-comparison test	Neutrophils vs control: p = 0.006; L-arginine + neutrophils vs control: p = 0.137; L-arginine vs control: p = 0.223; L-arginine + neutrophils vs neutrophils: p = 0.032; L-arginine vs neutrophils: p = 0.069; L-arginine + neutrophils vs L-arginine: p > 0.999
Figure 2C–D	8-oxo-dG immunofluorescence / oxidative nucleic-acid damage	n = 3 independent HUVEC cultures	Multiple fields per condition; fields were not treated as independent biological replicates	Shapiro–Wilk; Brown–Forsythe	Ordinary one-way ANOVA	Tukey's multiple-comparison test	Neutrophils vs control: p = 0.0002; L-arginine + neutrophils vs control: p = 0.3511; L-arginine vs control: p > 0.9999; L-arginine + neutrophils vs neutrophils: p = 0.0008; L-arginine vs neutrophils: p = 0.0002; L-arginine + neutrophils vs L-arginine: p = 0.3511
Figure 3A	Stable nitric oxide metabolites / NOx	n = 10 independent HUVEC cultures	Technical replicates averaged before statistical analysis	Shapiro–Wilk; Brown–Forsythe	Repeated-measures one-way ANOVA	Tukey's multiple-comparison test	Neutrophils vs control: p = 0.017; L-arginine + neutrophils vs control: p = 0.008; L-arginine vs control: p = 0.959; L-arginine + neutrophils vs neutrophils: p < 0.001; L-arginine vs neutrophils: p = 0.005; L-arginine + neutrophils vs L-arginine: p = 0.030
Figure 3B–C	Total eNOS and p-eNOS Ser1177 immunoblotting	n = 3 independent HUVEC cultures	Densitometry performed per independent culture; technical measurements averaged	Shapiro–Wilk; Brown–Forsythe	Ordinary one-way ANOVA	Tukey's multiple-comparison test	Total eNOS: no significant differences p = 0.9999; p-eNOS/eNOS: neutrophils vs control: p < 0 .001; L-arginine + neutrophils vs neutrophils: p < 0.001; catalase + neutrophils vs neutrophils: p < 0.001
Figure 4	Oxidant-sensitive DCF fluorescence	n = 6 independent HUVEC cultures	Technical replicates averaged before statistical analysis	Shapiro–Wilk; Brown–Forsythe	Repeated-measures one-way ANOVA	Tukey's multiple-comparison test	Neutrophils vs control: p = 0.010; L-arginine + neutrophils vs control: p = 0.875; L-arginine vs control: p = 0.461; L-arginine + neutrophils vs neutrophils: p = 0.002; L-arginine vs neutrophils: p < 0.001; L-arginine + neutrophils vs L-arginine: p = 0.626; catalase + neutrophils vs neutrophils: p = 0.004
Figure 5A	PECAM-1/CD31 immunofluorescence	n = 3 independent HUVEC cultures	Representative microscopic fields; fields not treated as biological replicates	Not applicable; qualitative endpoint	Not applicable; qualitative endpoint	Not applicable; qualitative endpoint	Qualitative structural support for HRP permeability; not included as a quantitative statistical endpoint
Figure 5B	HRP permeability assay	n = 6 independent HUVEC cultures	Technical replicates averaged before statistical analysis	Shapiro–Wilk; Brown–Forsythe	Repeated-measures one-way ANOVA	Tukey's multiple-comparison test	Neutrophils vs control: p = 0.001; L-arginine + neutrophils vs control: p = 0.161; L-arginine vs control: p = 0.990; L-arginine + neutrophils vs neutrophils: p = 0.031; L-arginine vs neutrophils: p < 0.001; L-arginine + neutrophils vs L-arginine: p = 0.435; catalase + neutrophils vs neutrophils: p = 0.009
Figure 6 / Table 1 / Supplementary Table S2	RT² Profiler PCR array	n = 3 independent HUVEC cultures per condition	GeneGlobe analysis performed using replicate 2^-ΔCt values	Not assessed gene-by-gene in GraphPad; GeneGlobe workflow used for raw p-value generation	Not applicable	GeneGlobe two-tailed Student's t-test for raw p-values; Benjamini–Hochberg FDR correction applied separately to each comparison across 84 target genes	HUVEC + neutrophils vs HUVEC: 18 raw p < 0.05, 5 FDR < 0.05; HUVEC + L-arginine + neutrophils vs HUVEC: 9 raw p < 0.05, 0 FDR < 0.05; HUVEC + L-arginine + neutrophils vs HUVEC + neutrophils: 0 raw p < 0.05, 0 FDR < 0.05; HUVEC + L-arginine vs HUVEC: 0 raw p < 0.05, 0 FDR < 0.05

Note: Unless otherwise stated, the experimental unit was the independent HUVEC donor. For each quantitative endpoint, all experimental conditions were generated in parallel from the same HUVEC donor IDs listed in Supplementary Table S1. Donor-matched analyses were performed using repeated-measures one-way ANOVA followed by Tukey's multiple-comparison test when applicable. For endpoints in which repeated-measures one-way ANOVA could not be applied in GraphPad Prism, ordinary one-way ANOVA followed by Tukey's multiple-comparison test was used, as indicated in the table. Technical replicates were averaged before statistical analysis and were not treated as independent biological replicates. For immunofluorescence-based endpoints, multiple microscopic fields were analyzed per condition but were not considered independent biological replicates. PECAM-1/CD31 immunofluorescence was assessed qualitatively as structural support for HRP permeability and was not included as a quantitative statistical endpoint. For the RT² Profiler PCR array, raw p-values were generated using the QIAGEN GeneGlobe two-tailed Student's t-test and were subsequently adjusted using the Benjamini–Hochberg false-discovery rate method across the 84 target genes for each comparison. Exact pairwise p-values are reported as multiplicity-adjusted p-values when derived from Tukey's multiple-comparison test.
