# Supplementary material for: L-Arginine Mitigates Neutrophil-Induced Endothelial Injury by Preserving Nitric Oxide Signaling and Redox Homeostasis
Source: Int J Mol Sci. 2026 Sep 19;27(18):8347. doi: 10.3390/ijms27188347 (PMC13607857; doi:10.3390/ijms27188347)
Supplement: Supplementary file 1 [file ijms-27-08347-s001.zip › Supplementary_Table_S4_Densitometric_quantification_of_WB.pdf]

Supplementary Table S4. Densitometric quantification of p-eNOS/eNOS and eNOS/GAPDH in HUVECs. Individual donor data

Study group	Donor ID	p-eNOS intensity (a.u.)	eNOS intensity (a.u.)	GAPDH intensity (a.u.)	p-eNOS/eNOS (a.u.)	eNOS/GAPDH (a.u.)
HUVEC control	HUVEC_06	98.589	97.877	66.325	1.007	1.476
HUVEC control	HUVEC_08	116.584	118.223	85.592	0.986	1.381
HUVEC control	HUVEC_10	73.953	67.843	90.563	1.090	0.749
HUVEC + L-arginine	HUVEC_06	96.334	99.877	71.156	0.965	1.404
HUVEC + L-arginine	HUVEC_08	111.127	126.141	101.892	0.881	1.238
HUVEC + L-arginine	HUVEC_10	64.220	68.794	92.675	0.934	0.742
HUVEC + neutrophils	HUVEC_06	40.496	98.473	74.621	0.411	1.320
HUVEC + neutrophils	HUVEC_08	57.486	110.674	99.081	0.519	1.117
HUVEC + neutrophils	HUVEC_10	37.492	73.826	94.885	0.508	0.778
HUVEC + L-arginine + neutrophils	HUVEC_06	94.333	98.473	78.098	0.958	1.261
HUVEC + L-arginine + neutrophils	HUVEC_08	101.474	113.074	113.999	0.897	0.992
HUVEC + L-arginine + neutrophils	HUVEC_10	54.298	67.936	104.762	0.799	0.648
HUVEC + CAT + neutrophils	HUVEC_06	90.092	112.800	80.178	0.799	1.407
HUVEC + CAT + neutrophils	HUVEC_08	108.847	113.128	100.585	0.962	1.125
HUVEC + CAT + neutrophils	HUVEC_10	52.108	62.936	101.612	0.828	0.619
